# COVID‐19 vaccine wastage in Thailand amidst vaccine inequity and pandemic‐to‐endemic transition

**DOI:** 10.1002/puh2.66

**Published:** 2023-02-01

**Authors:** Manatee Jitanan, Rapeeporn Tiamjan, Sarinporn Chaivisit, Pornchan Lojanasupareuk, Manas Ranjan Behera, Theerapon Phungdee, Wayu Waengkaew, Adriana Viola Miranda

**Affiliations:** ^1^ Department of Physical Education Faculty of Education Kasetsart University Bangkok Thailand; ^2^ Department of Public Health Faculty of Science and Technology Chiang Mai Rajabhat University Chiang Mai Thailand; ^3^ Department of Educational Technology Faculty of Education Kasetsart University Bangkok Thailand; ^4^ School of Public Health Kalinga Institute of Industrial Technology (KIIT) University Bhubaneswar India; ^5^ Global Health Focus Asia Bandung Indonesia

**Keywords:** COVID‐19, pandemic, pandemic‐to‐endemic transition, Thailand, vaccination, vaccine wastage

## Abstract

COVID‐19 vaccine wastage is an emerging issue globally. In Thailand, which has declared COVID‐19 to be an endemic disease, the risk of vaccine wastage lingers despite the remaining vaccine inequity. Although the country does not publicly report its vaccine wastage rate, a wastage incident has been reported. With the remaining secured vaccine doses that have yet to be delivered and Thailand's pandemic‐to‐endemic transition, Thailand should strategize to avoid vaccine wastage. This commentary highlights several factors that can potentially lead to vaccine wastage in Thailand: cold chain logistics issues, vaccine hesitancy, and unequal vaccine accessibility. The Thai government should continue its efforts in mitigating these issues to maximize its vaccination program.

## INTRODUCTION

COVID‐19 is a global public health threat that has overburdened the health‐care systems across countries. To attain herd immunity, vaccination programs have been conducted globally since 2021 [1]. However, COVID‐19 vaccine wastage continues to hamper this progress: Publicly available reports showed that approximately 158 million doses have been discarded as of July 2022. It is estimated that the actual number is much higher due to underreporting: Of the vaccine doses supplied globally, about 10% (1.1 billion doses) are estimated to have been wasted, with some countries reporting higher wastage rates [2, 3]. This is higher than the 5% general vaccine wastage recommended by the GAVI Alliance, as well as the 10% vaccine wastage rate it assumed for its COVID‐19 vaccination delivery [2, 3, 4]. Although the World Health Organization (WHO) does not advocate for a universal vaccine wastage level, considering differences in local outreach capabilities and other factors, it is recommended that vaccine wastage should be analyzed in relation to immunization coverage [4]. Considering the inequality of the global COVID‐19 vaccination rate, it is urgent to address vaccine wastage issues.

In Thailand, the first case of COVID‐19 was confirmed on 13 January 2020. Since then, as of 25 November 2022, there have been 4702,330 total confirmed cases of COVID‐19, with 33,106 deaths reported to WHO. The vaccination program, which started in June 2021, has achieved its goal of vaccinating 70% of Thai citizens [1, 5]. As a result of the widespread vaccination coverage, the lessening number of cases and the perceived herd immunity, starting on 1 October 2022, the Ministry of Public Health of Thailand downgraded COVID‐19 status from a dangerous communicable disease to an endemic disease subject to surveillance [6]. Coinciding with the changing designation of COVID‐19 by the Thai authorities, on 30 September 2022, the largest vaccination center in Thailand called the Bang Sue Injection Center was closed [7]. Several vaccination centers across Thailand have also announced their closure.

However, the perceived herd immunity continues to be threatened by the ongoing vaccine inequity. It is known that Thai ethnic minorities and other vulnerable groups in rural areas have less vaccine coverage compared to urban citizens. Two provinces on the south border of Thailand, Narathiwat and Pattani provinces, still have <50% of two‐dose vaccination coverage as of 23 November 2022 [8]. In addition, the emergence of new variants, such as Omicron and Omicron XBB, necessitates booster doses [1].

Despite these needs, vaccine wastage has occurred from unused vaccines: Although the exact number of expired vaccines within the country is not available to the public, vaccine expiration at the Bang Sue Injection Center was reported in 2021. This was soon followed by an announcement of a joint agreement between the Thai government and vaccine manufacturers to extend the shelf life of several vaccines (Pfizer, Moderna, and AstraZeneca) from 7 to 9 months [9]. Furthermore, the risk of vaccine wastage lingers as, despite the closure of Thai vaccination sites, Thailand is still expecting the delivery of secured vaccine doses. As of 26 November 2022, Thailand has secured doses that cover 337.7% of its population, of which 64% have arrived in the country and 57% have been administered to Thai citizens [10]. The government is also expanding its vaccination programs to cover younger age groups [11]. Without refining the Thai vaccination programs strategy, vaccine wastage will be an issue amidst vaccine inequity in the country. This commentary aims to analyze the current vaccine wastage situation in Thailand and its driving factors as well as provide recommendations to mitigate vaccine wastage and optimize its vaccination program.

## FACTORS ASSOCIATED WITH VACCINE WASTAGE

The WHO divides vaccine wastage into two types: closed vial wastage and open vial wastage. Closed vial wastage happens when vials have physical damage are not kept at the necessary temperature during storage or transportation, or when vaccines are out of expiration before they are opened. Open vial wastage happens because of spillage, physical damage to open vials, and expiry. It occurs when unused doses from multidose vials are thrown away [4].

Several factors are known to lead to vaccine wastage: vaccine characteristics, cold chain logistics issues, vaccine hesitancy, and unequal vaccine accessibility. In terms of vaccine characteristics, each COVID‐19 vaccine has a different risk of vaccine wastage: For instance, Pfizer/BioNTech vaccines are at a higher risk of closed vial wastage due to their low‐temperature requirements. Vaccines with a higher number of doses per vial, such as AstraZeneca, are at higher risk of open vial wastage. Limited cold chain logistics can also hinder the effective storage and transportation of these vaccines. Lastly, vaccine hesitancy and unequal vaccine accessibility can also lead to vaccine wastage as these issues limit vaccine uptake [4]. For instance, vaccine hesitancy in Hong Kong was estimated to cause up to 2 million vaccine waste [3]. Ineffective cold chain logistics and limited vaccine uptakes lead to the vaccines being unused and later expired, causing vaccine wastage. In this section, these risk factors are mapped within the Thai context.

### Cold chain logistics

There are seven vaccine types used in Thailand: Moderna, Pfizer, Johnson & Johnson, AstraZeneca, Sinopharm, Sinovac, and Serum Institute of India [1, 12]. These vaccines have different storage and transportation requirements, with several vaccines needing ultracold storage (e.g., −80°C for Pfizer). Before conducting its mass vaccination program, Thailand conducted assessments on its cold chain logistics and provided investments to its provinces and districts accordingly [13]. Since then, according to the Asian Development Bank in 2021, the traditional cold chain preparedness (2–8°C) of Thailand attains the maximum score of five. It is among the highest in the Asia Pacific. However, its ultracold chain preparedness is scored at 3/5 [14]. This is an issue for two reasons: First, the remaining undelivered vaccines include those requiring ultracold storage; second, the recently started Thai vaccination program for children aged 6 months to 4‐year old only authorized Pfizer as its vaccine; lastly, booster vaccination programs also require good quality logistics [11]. The Thai government is also distributing vaccines under a specific vendor‐managed inventory system that monitors vaccine utilization and wastage rates. However, the exact number of wastage is not reported to the public [13].

### Vaccine hesitancy

Vaccine hesitancy has been reported among Thai citizens since the early phase of the vaccination program: in 2021, 10%–40% hesitated to be vaccinated [15, 16]. Lack of vaccine acceptance has also been reported in rural areas, specifically among members of the hill tribes living in the border areas of Thailand and Myanmar, citing the need for more information about the vaccines. On the other hand, the available awareness programs are hindered by language barriers and communication issues experienced by the tribes, which has distinct languages and culture [17]. There were also beliefs that COVID‐19 would not impact people in remote areas [18]. Although this hesitancy seems to be addressed in 2022, vaccine hesitancy continues to be experienced by parents (58%). This is despite 96.7% of the parents having been vaccinated, although statistical analysis showed that having completed the initial vaccination (two‐dose) is a protective factor. Negative attitudes toward COVID‐19 and COVID‐19 vaccines, a history of refusing COVID‐19 vaccination for themselves and other vaccinations for children are associated with a higher risk of vaccine hesitancy [19]. This may limit the vaccine uptake in the recently launched vaccination drive for children, causing wastage of the vaccines allocated for the program.

### Unequal vaccine accessibility

As of 23 November 2022, 70% of the provinces of Thailand (54 out of 77) report >70% of two‐dose vaccination coverage. However, vaccination coverage is significantly lower in border provinces. This is especially a problem in south‐bordering provinces, of which two provinces have <50% coverage and one province reports <60% coverage. Mae Hong Son, a province bordering Myanmar, which is home to the fifth largest stateless population in Thailand, also reports <60% coverage [8]. Health‐care workers at Mae Hong Son Province reported that the storage requirement of the COVID‐19 vaccines is one of the main issues, as it is not always possible to deliver the vaccines to remote areas while keeping the cold temperature. It is known that the infrastructure in remote areas, such as roads, is very limited: For instance, reaching a village that is only 10–30 km from the main districts takes 2–3 h by car [18]. Nationality requirements have also hindered access to vaccination programs: In bordering provinces, tribe members are not always given Thai citizenship [17].

Considering most provinces have a decent vaccination coverage, vaccine supply delivery prioritizes remote areas. The government has collaborated with several United Nations (UN) bodies and nongovernmental organizations to improve vaccine accessibility [18, 20]. However, as some of these problems are policy‐based (such as citizenship and infrastructure development), the Thai government needs to provide more efforts to ramp up vaccination coverage in these bordering provinces. Without sufficient efforts, the remote areas prioritization may lead to vaccine wastage due to inoptimal vaccine uptake.

## RECOMMENDATIONS FOR PREVENTION

Addressing COVID‐19 vaccine wastage will enable the Thai government to maximize the usage of its vaccine supply to populations in need. To reduce the risk of COVID‐19 vaccine wastage in Thailand, the Thai government must address its cold chain limitations, vaccine hesitancy, and unequal vaccine accessibility. The Thai government should continue procuring cold chain logistics, particularly ultracold chain logistics. Periodic reporting of COVID‐19 vaccine wastage should be considered, either publicly or within the Thai health system. The vendor‐managed inventory system should be equipped with a program that can swiftly reallocate these vaccines following needs and expiry dates.

Vaccine awareness programs should be continued to improve the vaccine acceptance rate, with a focus on the rural population and parents. Culture‐sensitive approaches that address language barriers should be prioritized, particularly in the bordering provinces with <60% coverage. More research is needed to understand the reasons behind parents’ hesitancy to COVID‐19 vaccines despite being vaccinated. A specific campaign on vaccine efficacy, vaccine benefit, and vaccine safety for children should be conducted. Booster vaccination campaigns should also be continued to ensure good uptake within the community.

Vaccine accessibility in remote areas should be improved by investing in cold chain logistics and creating vaccination programs for non‐Thai citizens and the stateless population. Collaborations with the UN bodies and NGOs should be fostered. As a long‐term strategy, the Thai government should invest in the infrastructure of these areas.

## CONCLUSION

Amidst its pandemic‐to‐endemic transition, Thailand is facing the risk of COVID‐19 vaccine wastage. Several factors can potentially cause this issue: cold chain logistics issues, vaccine hesitancy, and unequal vaccine distribution. To solve this issue, the Thai government needs to ramp up its efforts that have successfully led to its vaccination target. Cold chain procurement, awareness programs, and investment in remote areas should be prioritized.

## AUTHOR CONTRIBUTIONS

*Conceptualization; data curation; formal analysis; writing—original draft; writing—review and editing*: Manatee Jitanan. *Data curation; formal analysis; writing—original draft*: Rapeeporn Tiamjan. *Data curation; formal analysis; writing—original draft*: Sarinporn Chaivisit. *Data curation; formal analysis; writing—original draft*: Pornchan Lojanasupareuk. *Data curation; formal analysis; writing—original draft*: Manas Ranjan Beher. *Data curation; formal analysis; writing—original draft*: Theerapon Phungdee. *Data curation; formal analysis; writing—original draft*: Wayu Waengkaew. *Supervision; writing—review and editing*: Adriana Viola Miranda.

## CONFLICT OF INTEREST STATEMENT

AVM is an editorial board member of the journal. She was excluded and blinded from all stages of the peer review of this manuscript.
